# Screening pregnant women for suicidal behavior in electronic medical records: diagnostic codes vs. clinical notes processed by natural language processing

**DOI:** 10.1186/s12911-018-0617-7

**Published:** 2018-05-29

**Authors:** Qiu-Yue Zhong, Elizabeth W. Karlson, Bizu Gelaye, Sean Finan, Paul Avillach, Jordan W. Smoller, Tianxi Cai, Michelle A. Williams

**Affiliations:** 1 Department of Epidemiology, Harvard T.H. Chan School of Public Health, Boston, MA 02115 USA; 2 Department of Medicine, Division of Rheumatology, Immunology and Allergy, Brigham and Women’s Hospital and Harvard Medical School, Boston, MA USA; 3 Children’s Hospital Informatics Program, Boston Children’s Hospital, Boston, MA USA; 4 Department of Biomedical Informatics, Harvard Medical School, Boston, MA USA; 5 Psychiatric and Neurodevelopmental Genetics Unit, Massachusetts General Hospital, Boston, MA USA; 6 Department of Biostatistics, Harvard T.H. Chan School of Public Health, Boston, MA USA

**Keywords:** Natural language processing, Electronic medical records, Pregnancy, Suicidal behavior, Screening, Diagnostic codes, Clinical notes

## Abstract

**Background:**

We examined the comparative performance of structured, diagnostic codes vs. natural language processing (NLP) of unstructured text for screening suicidal behavior among pregnant women in electronic medical records (EMRs).

**Methods:**

Women aged 10–64 years with at least one diagnostic code related to pregnancy or delivery (*N* = 275,843) from Partners HealthCare were included as our “datamart.” Diagnostic codes related to suicidal behavior were applied to the datamart to screen women for suicidal behavior. Among women without any diagnostic codes related to suicidal behavior (*n* = 273,410), 5880 women were randomly sampled, of whom 1120 had at least one mention of terms related to suicidal behavior in clinical notes. NLP was then used to process clinical notes for the 1120 women. Chart reviews were performed for subsamples of women.

**Results:**

Using diagnostic codes, 196 pregnant women were screened positive for suicidal behavior, among whom 149 (76%) had confirmed suicidal behavior by chart review. Using NLP among those without diagnostic codes, 486 pregnant women were screened positive for suicidal behavior, among whom 146 (30%) had confirmed suicidal behavior by chart review.

**Conclusions:**

The use of NLP substantially improves the sensitivity of screening suicidal behavior in EMRs. However, the prevalence of confirmed suicidal behavior was lower among women who did not have diagnostic codes for suicidal behavior but screened positive by NLP. NLP should be used together with diagnostic codes for future EMR-based phenotyping studies for suicidal behavior.

**Electronic supplementary material:**

The online version of this article (10.1186/s12911-018-0617-7) contains supplementary material, which is available to authorized users.

## Background

Suicide, a devastating event, is one of the leading cause of maternal deaths during pregnancy and the peripartum period [1, 2]. Early detection of pregnant women with nonfatal suicidal thoughts and behavior (hereafter referred to as suicidal behavior) presents an important opportunity for directing suicide prevention efforts to those at high risk for suicide and, therefore, can help to prevent maternal mortality [3–5]. However, low-cost, highly scalable methods to identify suicidal behavior are lacking. To date, studies have primarily relied on the International Classification of Diseases (ICD) billing codes using administrative or claims data to identify instances of suicidal behavior [5–9]. Suicidal behavior is often “under-coded” with only a small proportion of suicidal cases being detected by the ICD codes among all suicidal cases (i.e., low sensitivity) [10–13]. For example, a systematic review [9] reported that the sensitivity of one widely used ICD-9 code category, suicide and self-inflicted injury (E950–E959), ranged from 13.8 to 65%. Using a large primary care database from the United Kingdom (UK), Thomas et al. [12] reported that the use of diagnostic codes to detect suicidal cases missed approximately three-quarters of the cases. The reported low sensitivity of billing codes for identifying suicidal behavior implies that a sizable portion of suicidal cases may be missed when case-finding relies on ICD codes alone. Therefore, expanded data collection methods for suicidal behavior are urgently needed to provide a foundation for prevention efforts [9, 14].

The increasing utilization of electronic medical records (EMRs) has provided unprecedented opportunities for identifying pregnant women with suicidal behavior. EMRs contain a ready repository of clinical and phenotypic information consisting of structured and unstructured data that can enable low-cost population-based studies [15, 16]. Structured data are entered by “clicking” on choices of lists, forms, or templates, including demographic data, laboratory test results, and diagnostic billing codes such as the aforementioned ICD codes [16–18]. Unstructured data—clinical data extracted from free-text such as physicians’ notes and radiology reports—offers a valuable resource for defining clinical phenotypes [19–22]. The automated examination of a large volume of clinical notes requires the use of natural language processing (NLP) [23], a field of computational linguistics that allows computers to extract relevant information from unstructured human language [22]. NLP has been used successfully to identify patient cohorts for different phenotypes including treatment resistant depression, bipolar disorder, cerebral aneurysms, rheumatoid arthritis, Crohn’s disease, ulcerative colitis, and diabetes [15, 23–32]. However, very few studies have used NLP to identify suicidal behavior in EMRs [10, 33, 34], and no study has reported any classification algorithm that is highly predictive of suicidal behavior.

Because of the low prevalence of suicidal behavior [4, 35], developing a phenotyping algorithm using the full EMR population would likely result in low positive predictive values (PPV) [36]. To address this, we first screened for patients with medical record information (structured or unstructured) suggestive of suicidal behavior and excluding those with no evidence of suicidal behavior [36]. The patients who screened positive for suicidal behavior would serve as a highly sensitive datamart and then can be used to develop highly predictive classification algorithms for suicidal behavior. Here, using EMRs from a large healthcare system (Partners HealthCare), we demonstrate that using diagnostic codes together with NLP can more effectively screen for pregnant women with a higher potential of suicidal behavior. We also compare the characteristics of patients identified by these two methods.

## Methods

### Data source and study population

We extracted data from the Partners HealthCare System Research Patient Data Registry (RPDR). The RPDR is a centralized clinical data warehouse for 4.6 million patients from two large academic medical centers (Massachusetts General Hospital [MGH] and Brigham and Women’s Hospital [BWH]), as well as community and specialty hospitals in the Boston area. The RPDR includes structured and unstructured EMR information, including socio-demographic data, vital signs, laboratory and test results, problem list entries, prescribed medications, billing codes, and clinical notes for healthcare services provided within the system [37]. The Institutional Review Board of Partners HealthCare (Protocol Number: 2016P000775/BWH) and Harvard T.H. Chan School of Public Health (Protocol Number: IRB16–0899) approved all aspects of this study.

**Fig. 1:**
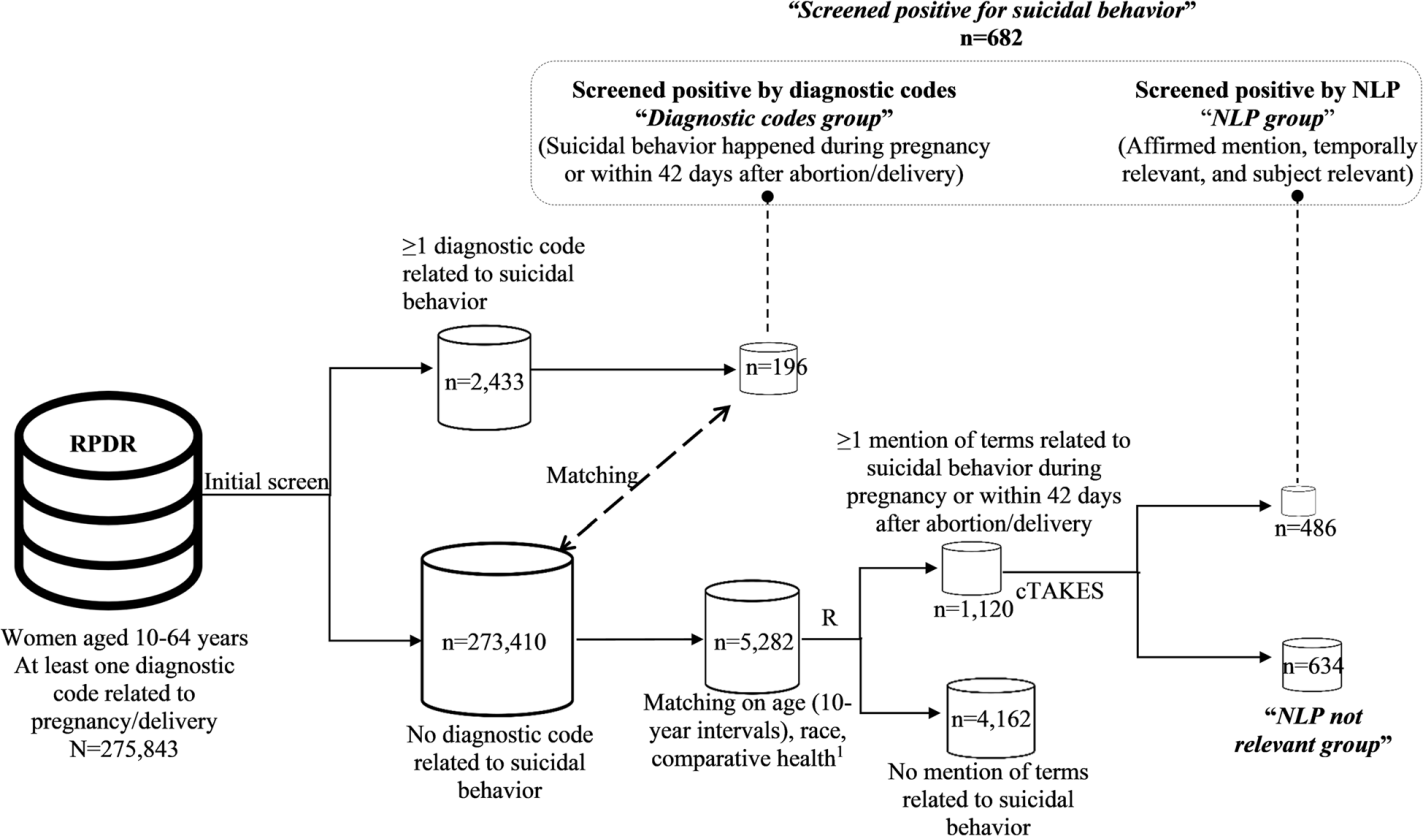
Screening for suicidal behavior using diagnostic codes vs. NLP among pregnant women. Abbreviations: Natural Language Processing (NLP). ^a^ Comparative health: the total number of facts which included diagnostic codes for diseases, medications, and specific test results from hospital visits for each patient; it can be used as a proxy for healthcare utilization

We initially identified women aged 10–64 years with at least one diagnostic code related to pregnancy or delivery (International Classification of Diseases-10 [ICD-10]: Z3A.*, O0.*- O9.*; ICD-9: 640.*- 679.*, V22.*, V23.*, V24.*, V27.*, V28.*; Diagnosis-Related Group [DRG]: 370–384) in the EMRs from January 1, 1996 to March 31, 2016, totaling 275,843 women (hereafter referred to as “datamart”) included in the datamart (Fig. 1).

### Suicidal behavior screened positive by diagnostic codes

We first screened for suicidal behavior based on diagnostic codes including the ICD codes and the Longitudinal Medical Record (LMR) codes. The LMR codes were assigned to problem list conditions in the ambulatory EMR system used across Partners HealthCare System. (Additional file 1: Table S1). In addition to the explicit diagnostic codes for suicidal ideation (e.g., ICD-9 V62.84) and suicide attempt (e.g., ICD-9 E95*), we also included additional sets of ICD code categories (poisoning by analgesics, antipyretics, and antirheumatics; poisoning by sedatives and hypnotics; and poisoning by psychotropic agents) with positive predictive value ≥0.8 for suicidal behavior, based on a previous study [4]. Among the 275,843 women with at least one diagnostic code related to pregnancy or delivery, 2433 women had at least one diagnostic code related to suicidal behavior, of which 196 had a diagnostic code that occurred during pregnancy, or within 42 days after abortion or delivery [38]. These 196 women, who screened positive for suicidal behavior based on diagnostic codes, hereafter will be referred to as the “diagnostic codes group” (Fig. 1).

### Suicidal behavior screened positive by NLP-processed clinical notes

Among the 273,410 women without any diagnostic codes related to suicidal behavior, we randomly sampled a subset of women (*N =* 5880) who were matched for age (10-year intervals), race, and comparative health with the diagnostic codes group using a 1:30 matching ratio. The reason we chose the 1:30 ratio for subsequent NLP was twofold: (1) to provide a sample size that was large enough for a general view of distributions of CUIs, and (2) to minimize the NLP processing time. Comparative health, a proxy for healthcare utilization, was defined as the total number of observations in the medical records which included diagnostic codes for diseases, medications, and specific test results from hospital visits for each patient [39]. To comply with the IRB, Partners HealthCare employees (*N =* 598) were excluded, leaving 5282 women in the matched set. We then searched women’s clinical notes and identified 1120 (21.2%) women with at least one mention of the terms related to suicidal behavior [12] (Additional file 1: Table S2) during pregnancy or within the 42 days after abortion or delivery [38].

We further processed the clinical notes of the 1120 women using the clinical Text Analysis and Knowledge Extraction System (cTAKES 3.2.3, http://ctakes.apache.org/) [40]. Based on the Unstructured Information Management Architecture (UIMA), cTAKES is a comprehensive clinical NLP tool that processes clinical notes and identifies terms. cTAKES maps the terms to a subset of the Unified Medical Language System (UMLS) Metathesaurus [41], the Systemized Nomenclature of Medicine-Clinical Terms (SNOMED-CT) [42], and assigns each term a UMLS concept unique identifier (CUI). cTAKES also extracts qualifying attributes (including negation, temporality, and subject status) associated with each CUI. As determined by cTAKES negation module [40], each CUI can be either affirmed (e.g., “patient reports feeling suicidal”) or negated (e.g., “suicidal behavior: none”). Affirmed CUIs were considered as relevant for this analysis. cTAKES has a temporality module, *DocTimeRel* (Document Time Relation), to discover the temporal relation between a term and the document creation time [24]. The values for *DocTimeRel* include “before” (e.g., “patient attempted suicide when she was 14”), “after” (e.g., “She would not consider suicide an option if symptoms were to arise”), “overlap” (e.g., “patient states that she wants to kill herself”), and “before/overlap” (terms that started before document creation time and continue to the present [e.g., “patient endorses passive suicidal ideation since the birth of her baby”]). Terms tagged as “overlap” or “before/overlap” were considered as temporally relevant for this analysis. The *Subject* module indicates whether the patient or someone else (e.g., “mom attempted suicide”) experiences the event. The values for the *Subject* module include “patient,” “family member,” “other,” and “null.” [43] The terms tagged as “patient” were considered as subject relevant for this analysis.

We created an expert-defined list of CUIs considered relevant to suicidal behavior (Additional file 1: Table S3). We included the distributions of attributes of the CUIs relevant to suicidal behavior in Additional file 1: Table S4.

To compensate for errors introduced by the NLP system, we calculated the proportion of affirmed, temporally relevant, and subject relevant CUIs related to suicidal behavior among all CUIs related to suicidal behavior for each woman and selected women with proportions that were greater than or equal to 0.25. This threshold was determined empirically with an aim to decrease false positives, while maintaining relatively low false negatives. From the NLP-processed clinical notes, we identified 486 pregnant women (hereafter referred to as the “NLP group”) with CUIs related to suicidal behavior. Of note, the NLP group was screened positive by both term mentions related to suicidal behavior and cTAKES. The remainder (*N* = 634) who had at least one mention of the terms related to suicidal behavior during pregnancy or within the 42 days after abortion or delivery, but were not screened positive by the NLP are referred to as the “NLP not relevant group.”

### Reference group

We randomly sampled a subset of women aged 10–64 years with at least one diagnostic code related to pregnancy or delivery as the reference group. The reference group was matched with comparative health [39] for the diagnostic codes group using a 1:100 matching ratio. Since we did not need to process the clinical notes for reference group, we included a relatively larger sample size. After excluding Partners HealthCare employees, 17,183 women were included in the reference group.

### Chart review to obtain estimates for prevalence of confirmed suicidal behavior

After the screening process, one of the authors (QYZ) manually reviewed the clinical notes for random samples of (1) 50 women from the diagnostic codes group (*N* = 196); (2) 100 women from the NLP group (*N* = 486); (3) 100 women from the NLP not relevant group (N = 634); and (4) 100 women who had neither diagnostic codes nor term mentions related to suicidal behavior (*N* = 4162). Based on the Columbia Classification Algorithm of Suicide Assessment (C-CASA), the reviewer assigned each woman a classification of either “with” or “without suicidal behavior” [44]. Women who had (1) completed suicide, (2) suicide attempt, (3) preparatory acts toward imminent suicidal behavior, or (4) suicidal ideation were considered as “with” suicidal behavior.

### Statistical analysis

We compared the demographic and provider characteristics of pregnant women screened positive for suicidal behavior by the diagnostic codes versus NLP during encounters with suicidal behavior. We examined the distributions of demographic characteristics between pregnant women screened positive for suicidal behavior by the diagnostic codes versus NLP using the Chi-square test for categorical variables and Student’s *t*-test for continuous variables. We reported the proportions of women who received diagnoses of psychiatric comorbidities at least once during or before the most recent encounter with suicidal behavior. Psychiatric comorbidities were defined using the ICD codes in Additional file 1: Table S5. All analyses were done using R [45].

## Results

We identified 682 pregnant women who screened positive for suicidal behavior, of whom 196 (28.73%) were identified by diagnostic codes and 486 (71.26%) were identified by NLP. Based on manual chart review, the prevalence of confirmed suicidal behavior in women screened positive (PPV) by the diagnostic codes and by NLP in women without the diagnostic codes were 76.00 and 30.00%, respectively. The estimated number of confirmed suicidal behavior among the screen positive groups by the diagnostic codes and NLP would be 149 and 146, respectively. The prevalence of confirmed suicidal behavior was 1.00% among the NLP not relevant group. The prevalence of confirmed suicidal behavior was 0.00% among women who had neither diagnostic codes nor term mentions related to suicidal behavior. The approximate estimated prevalence of suicidal behavior in the reference group would be 2.76% (486 × 0.3/5282).

The demographic characteristics of women who screened positive for suicidal behavior by the diagnostic codes and NLP, respectively, are presented in Table 1. Compared with the NLP group, the diagnostic codes group was less likely to be Hispanic (33.33% vs. 28.57%), be married/common-law married/partnered (29.63% vs. 21.43%), report religious affiliation as Christian (45.47% vs. 38.27%), and have private insurance (44.65% vs. 32.14%); these women were more likely to be Black or African American (16.46% vs. 20.92%), be single (65.02% vs. 71.43%), and be insured by Medicaid (43.21% vs. 49.49%) and Medicare (6.17% vs. 9.18%).

**Table 1:** Demographic characteristics of pregnant women screened positive for suicidal behavior by diagnostic codes vs. NLP

Characteristics	Diagnostic codes (*N* = 196)		NLP^a^(*N* = 486)		*P-*values^b^	Reference group^c^(*N* = 17,183)	
	n	%	n	%		n	%
Age at the most recent pregnancy with suicidal behavior^d^	26.8 (6.9)		26.4 (6.2)		0.46	35.7 (8.3)^e^	
Age at the most recent pregnancy with suicidal behavior^d^					0.14		
< 16	4	2.04	2	0.41		38	0.22
[16, 18)	8	4.08	27	5.56		65	0.38
[18, 20)	16	8.16	53	10.91		205	1.19
[20, 35)	141	71.94	352	72.43		7594	44.19
≥ 35	27	13.78	52	10.70		9281	54.01
Language					0.51		
English	168	85.71	407	83.74		14,844	86.39
Spanish	21	10.71	66	13.58		1537	8.94
Other	7	3.57	13	2.67		802	4.67
Race/ethnicity					0.20		
Asian	7	3.57	8	1.65		1036	6.03
Black or African American	41	20.92	80	16.46		1837	10.69
Hispanic	56	28.57	162	33.33		2677	15.58
White	87	44.39	211	43.42		10,413	60.60
Other/Not recorded	5	2.55	25	5.14		1220	7.10
Religion					0.34		
Christian	75	38.27	221	45.47		5904	34.36
Catholic	63	32.14	162	33.33		6627	38.57
Islamic	5	2.55	8	1.65		451	2.62
Jewish	1	0.51	6	1.23		676	3.93
No preference/None	27	13.78	49	10.08		1275	7.42
Other/Unknown/Not recorded	25	12.76	40	8.23		2250	13.09
Marital status					0.15		
Married/Partner/Common law	42	21.43	144	29.63		10,816	62.95
Single	140	71.43	316	65.02		4675	27.21
Separated/Divorced/Widowed	9	4.59	15	3.09		1000	5.82
Other/Unknown	5	2.55	11	2.26		692	4.03
Vital status					0.04		
Deceased with date of death	2^f^	1.02	6^f^	1.23		168	0.98
Deceased with date of death unknown	4	2.04	1	0.21		35	0.20
Not reported as deceased	190	96.94	479	98.56		16,980	98.82
Veteran					0.12		
No	169	86.22	421	86.63		14,810	86.19
Yes	3	1.53	1	0.21		75	0.44
Unknown	24	12.24	64	13.17		2298	13.37
Insurance					0.03		
Medicaid	97	49.49	210	43.21		3097	18.02
Medicare	18	9.18	30	6.17		531	3.09
Private Insurance	63	32.14	217	44.65		12,415	72.25
Self-pay	8	4.08	11	2.26		488	2.84
Other	10	5.10	18	3.70		652	3.79

Abbreviations: Natural language processing (*NLP*)

^a^Randomly sampled from women aged 10–64 years with at least one diagnostic code related to pregnancy or delivery, matching on age, race, comparative health with women screened positive for suicidal behavior by diagnostic codes using a 1:30 matching ratio

^b^For continuous variables, *P*-value was calculated using the Student’s t test; for categorical variables, *P*-value was calculated using the Chi-square test.

^c^Randomly sampled from women aged 10–64 years with at least one diagnostic code related to pregnancy or delivery, matching on comparative health with women screened positive for suicidal behavior by diagnostic codes using a 1:100 matching ratio

^d^Mean (Standard deviation)

^e^Age at most recent date with diagnostic codes related to pregnancy or delivery

^f^None of the deaths occurred within 183 days after suicidal behavior

Table 2 shows provider characteristics for participants’ encounters (inpatient or outpatient visits) with suicidal behavior. For encounters with suicidal behavior, more than two-thirds of women in the diagnostic codes group (69.39%) visited the Emergency Department, whereas only 17.49% of women in the NLP group visited the Emergency Department. The proportions of women screened positive for suicidal behavior treated in an inpatient setting was higher among those in the diagnostic codes group (39.29%), as compared with those in the NLP group (19.55%).

**Table 2:** Provider characteristics at encounters with suicidal behavior of pregnant women screened positive for suicidal behavior by diagnostic codes vs. NLP

Characteristics	Diagnostic codes (N = 196)		NLP (N = 486)		Reference group^a^(N = 17,183)	
	n	%	n	%	n	%
Hospitals (ever)						
Massachusetts General Hospital	105	53.57	249	51.23	12,678	73.78
Brigham and Women’s Hospital	95	48.47	250	51.44	12,800	74.49
Faulkner Hospital	0	0.00	1	0.21	4263	24.81
North Shore Medical Center	0	0.00	11	2.26	2659	15.47
Newton-Wellesley Hospital	0	0.00	4	0.82	5643	32.84
Spaulding Rehabilitation Hospital	0	0.00	2	0.41	1199	6.98
McLean Hospital	0	0.00	0	0.00	283	1.65
Clinics (ever)						
Emergency	136	69.39	85	17.49	9749	56.74
Psychiatry/Mental health/Behavioral health	7	3.57	221	45.47	2605	15.16
Obstetrics and Gynecology	12	6.12	18	3.70	11,386	66.26
Pediatrics	4	2.04	125	25.72	1253	7.29
Inpatient/outpatient (ever)						
Inpatient	77	39.29	95	19.55	14,116	82.15
Outpatient	157	80.10	407	83.74	17,055	99.26
Not recorded	0	0.00	46	9.47	9762	56.81

Abbreviations: Natural language processing (NLP)

^a^Provider characteristics during lifetime (ever)

Psychiatric comorbidities were common among women with suicidal behavior (Table 3). Women screened positive for suicidal behavior by the diagnostic codes had higher psychiatric comorbidities including depression, schizophrenia, bipolar disorder, post-traumatic stress disorder (PTSD), and substance abuse. The distribution of care providers according to clinical specialties (Department of Psychiatry/Mental Health/Behavioral Health and Emergency Department) were similar across psychiatric comorbidities (Table 3).

**Table 3:** Psychiatric comorbidities of pregnant women screened positive for suicidal behavior by diagnostic codes vs. NLP

Psychiatric Comorbidities	Diagnostic codes (N = 196)		NLP (N = 486)		Reference group (N = 17,183)	
	n	%	n	%	n	%
Psychiatric Comorbidities						
Depression	171	87.24	353	72.63	5150	29.97
Schizophrenia	10	5.10	10	2.06	95	0.55
Bipolar	45	22.96	40	8.23	489	2.85
PTSD	58	29.59	76	15.64	590	3.43
Substance abuse	102	52.04	173	35.60	2332	13.57
Anxiety	100	51.02	259	53.29	5184	30.17
Psychiatric comorbidities at encounters to Department Psychiatry/Mental Health/Behavioral Health (ever)						
Depression	93	47.45	180	37.04	1536	8.94
Schizophrenia	7	3.57	4	0.82	43	0.25
Bipolar	18	9.18	26	5.35	234	1.36
PTSD	29	14.80	44	9.05	312	1.82
Substance abuse	43	21.94	36	7.41	177	1.03
Anxiety	30	15.31	113	23.25	1058	6.16
Psychiatric comorbidities at encounters to Emergency Department (Ever)						
Depression	128	65.31	80	16.46	608	3.54
Schizophrenia	6	3.06	2	0.41	22	0.13
Bipolar	25	12.76	8	1.65	76	0.44
PTSD	28	14.29	9	1.85	54	0.31
Substance abuse	74	37.76	57	11.73	652	3.79
Anxiety	51	26.02	42	8.64	660	3.84

Abbreviations: Natural language processing (*NLP*), post-traumatic stress disorder (*PTSD*)

## Discussion

We demonstrated that the use of NLP along with term search substantially improved the sensitivity of screening suicidal behavior among pregnant women from a large EMR system. More than two-thirds of potential suicidal behavior and nearly half of confirmed suicidal behavior would have been missed if screening had relied solely on ICD codes. However, we observed that the PPV of NLP, the probability that a suicidal case identified by NLP was truly suicidal, was lower (30.00%) as compared to the diagnostic codes (76.00%). We found that women in the diagnostic codes group had more risk factors for suicidal behavior [46], including low socioeconomic status, being single, and psychiatric comorbidities as compared with those women in the NLP diagnostic group.

Prior studies have attempted to identify patients with suicidal behavior in unstructured clinical notes. Using the UK Clinical Practice Research Datalink, Thomas et al. found that searching for terms related to suicide in general practice consultation records identified 10.7% of the suicidal cases that were missed by ICD diagnostic codes [12]. Anderson et al. [33] processed the History of Present Illness notes of 15,761 patients with at least one diagnostic code of depression in primary care clinical organizations. A rule-based NLP system was developed to search for positive mention or negation of suicidal behavior using a list of terms related to suicidal behavior. The proportion of patients with corresponding ICD diagnostic codes indicating suicidal ideation and suicide attempt in the notes were 3% and 19%, respectively. Haerian et al. [10] used an NLP tool, the Medical Language Extraction and Encoding System (MedLEE), to identify suicidal behavior in the EMRs for pediatric and adult inpatients. Of note, they used a list of CUIs with a specific focus on suicidal behavior by drug overdose, which was different from the CUI list we used in our study. In their study, 469 potential cases were identified by the ICD diagnostic codes, and 4087 were identified by the NLP algorithm after filtering out CUIs that were negated or associated with family history. The intersection of both ICD diagnostic codes and the NLP algorithm identified 260 potential cases. The positive predictive values for the ICD diagnostic codes and the NLP algorithm were similar (55% for ICD and 60% for NLP). Despite the different NLP tools used across EMR systems, these results consistently suggested that suicidal behavior was often documented in clinical notes without being assigned any diagnostic codes that were designed for billing purposes. Suicidal behavior is a complex phenotype coupled with many psychosocial problems, where clinical notes are often used to capture the complexity and diagnostic uncertainty [47, 48]. Incorporating information from unstructured clinical notes through NLP in our study, we were able to screen a significant number of patients with potential suicidal behavior that would otherwise not be found using structured data alone. However, the PPV of NLP used in the current study was lower than that of the diagnostic codes. Nonetheless, we identified a comparable number of suicidal cases (149 for diagnostic codes vs. 146 for NLP) when using only a subsample of women (5880 out of 273,410) without any diagnostic codes related to suicidal behavior for NLP. Despite the low PPV of NLP, considering the large number of pregnant women without diagnostic codes related to suicidal behavior (*N* = 273,410) and the fact that suicidal behavior was often documented in clinical notes, we maintain that NLP procedures may be used to identify more suicidal cases. Therefore, for future studies using EMR-based phenotyping for suicidal behavior, an optimal approach to increase screening sensitivity may best involve combining the application of NLP procedures with the diagnostic codes.

Only 30% of the women who screened positive for suicidal behavior by NLP were confirmed to be suicidal by chart review (PPV = 0.30). A large proportion of women who were not suicidal were screened positive for suicidal behavior by NLP. Similar to one previous study [10], the majority of the false positives came from the incorrect qualifying attributes based on our error analysis by manual review of the clinical notes from 100 women in the NLP group, in particular, negation associated with CUIs. Negation is a well-known challenge for processing unstructured clinical notes [49]. One study showed that approximately half of the conditions indexed in dictated reports were negated [50, 51]. For suicidal behavior, clinicians are likely to document both the presence and absence of suicidal behavior [10]. In the Partners HealthCare EMRs, we observed a major negation structure for suicidal behavior: terms related to suicidal behavior were followed by a colon and a negation word without any sentence punctuation (e.g., “suicidal behavior: none,” “suicidal behavior: none reported,” and “suicidal behavior: denied”) (Additional file 1: Table S6). However, the standard cTAKES negation module NegEx [40, 52], a regular expression pattern matching algorithm that searches for predefined negation words around terms [53] was initially trained using the Intensive Care Unit discharge summaries [52], and is not able to recognize such negation structure [54]. Consequently, a considerable number of suicidal behavior terms that were negated were incorrectly identified as “affirmed.” Further enhancement of the negation algorithm with training data pertaining specifically to suicidal behavior is required to decrease the false positives [49, 55]. Other common reasons leading to cTAKES miscoding women without suicidal behavior as suicidal (Additional file 1: Table S6) included (1) incorrect recognition of “before” as “overlap” by the *DocTimeRel* module (e.g., *DocTimeRel* module treated history of suicidal behavior as current suicidal behavior: “Suicide attempt/gesture: history of, hospitalized inpatient psych unit for suicide attempt in 1996”); (2) incorrect recognition of “family member” as “patient” by the *Subject* module (e.g., *Subject* module treated the suicidal behavior of patient’s father as patient’s: “Pt also identifies strongly with father, who was often aggressive toward others and threatened suicide”); (3) failure to identify section titles (e.g., “Suicidal Behavior Hx of Suicidal Behavior:”) that do not describe the behavior of patients; and (4) failure to handle hypothetical conditions that temporally are neither recent nor historical (e.g., “If she has significant side effects from it such as lethargy/depression/irritability/suicidal thought, we will change it to LTG.”).

We found that women in the diagnostic codes group had different characteristics as compared to women in the NLP group. On the one hand, these differences could be due to the lower prevalence of confirmed suicidal behavior in the NLP group. Therefore, developing highly predictive classification algorithms is needed for the NLP group. On the other hand, the differences between women screened positive for suicidal behavior by the diagnostic codes and NLP suggest that the two groups may differ with respect to the degree of suicide intent, methods used, and subsequent clinical management. Because a larger proportion of women screened positive by the diagnostic codes received inpatient care and were seen in the Emergency Department, they were likely to present as more severe cases of suicidal behavior with high suicide intent [56], requiring hospital admission and immediate care. In addition, the diagnostic codes for suicidal ideation (ICD-9: V62.82) were not used until October 2005 when the codes were introduced. Even after the codes became available, one study showed that suicidal ideation was less likely to be coded than suicide attempt [33]. These two factors (i.e., source of inpatient care and timing of availability of diagnostic codes) might have contributed to a disproportionate representation of more severe cases of suicidal behavior in the diagnostic codes group. In this scenario, women screened positive by the diagnostic codes may be a more relevant cohort for assessing patients at high risk for completed suicide [57], whereas women screened positive by NLP may be more relevant for investigating early identification of high-risk groups and suicide prevention interventions. Another possibility for the observed differences in characteristics, especially for psychiatric comorbidities, between the diagnostic codes group and the NLP group could be due to differential bias in coding: women with more risk factors were more likely to be coded for suicidal behavior.

There are several limitations of this study. First, the prevalence of confirmed suicidal behavior among women screened positive by NLP was only 30%. However, given the purpose of our study, which was to screen pregnant women with a higher potential of suicidal behavior and to develop a highly sensitive datamart for suicidal behavior, this low PPV might be tolerated. Nevertheless, using this highly sensitive datamart for suicidal behavior, future development of accurate classification algorithms using different machine learning techniques [58, 59] is clearly needed to identify true cases of suicidal behavior. Second, given the small sample size of women screened positive for suicidal behavior by the diagnostic codes, we did not further classify patients according to subtypes of suicidal behavior such as suicidal ideation and suicide attempt. Third, given that a woman was considered as screened positive for suicidal behavior only if she was screened positive by both term mention and NLP by cTAKES, it is possible that we might miss some women who did not pass the screening by term mentions related to suicidal behavior but would have been considered as screened positive by cTAKES. Fourth, we used 20 years of data from a single urban-regional EMR system that did not include patient visits outside this geographical area, time period, or network of hospitals. The generalizability of our results to patients in other healthcare systems may vary depending on the informatics infrastructure and local documentation practices [26]. Fifth, we focused on extracting facts expressed directly in the clinical notes (i.e., terms of suicidal behavior) using NLP. However, beyond extracting these basic facts, further research in studying other linguistic features, such as sentiment expressed in clinical notes (e.g., positive and negative emotions), and capturing the meaning of texts (e.g., word embedding [60–62]), may also be beneficial in identifying suicidal patients [63–67].

## Conclusion

Our results illuminated the advantage of using NLP along with term search in EMRs to screen pregnant women for a complex, rare psychiatric phenotype. NLP substantially improved the sensitivity of screening for suicidal behavior in an obstetric population. We captured a group of pregnant women with potential suicidal behavior otherwise not reflected in the structured data. We also highlight the challenges of using NLP in screening pregnant women for suicidal behavior. Of note, NLP had lower PPV as compared with diagnostic codes. Improvement in the cTAKES modules, especially the negation module, may help to increase the PPV. For future studies using EMR-based phenotyping for suicidal behavior, an optimal approach may include combining NLP procedures with the diagnostic codes.

Our approach is the first to examine the large-scale use of NLP in suicidal behavior among pregnant women. The current study in our population of pregnant women was particularly challenging given the rarity of suicidal behavior, the stigma attached, the complexity of phenotypic assessment, and the historical misconception of the protective role of pregnancy in suicidal behavior [3, 68]. Because pregnancy is a time when women have frequent interactions with the healthcare system, EMR-based identification of pregnant women with suicidal behavior may be useful for future genetic, epidemiological, and clinical studies, presenting a valuable opportunity for healthcare providers to intervene promptly [5, 69].

## Additional file

Additional file 1: **Table S1.** International Classification of Disease (ICD) codes and other diagnostic codes used to screen suicidal behavior. **Table S2.** Terms used to screen suicidal behavior in clinical notes. **Table S3.** Concept Unique Identifiers (CUIs) related to suicidal behavior. **Table S4.** Distributions of attributes of the Concept Unique Identifiers (CUIs) related to suicidal behavior among 1120 women. **Table S5.** International Classification of Disease (ICD) codes used to define psychiatric comorbidities. **Table S6**. Error analysis of false positive results from cTAKES to screen for suicidal behavior. (DOCX 36 kb)

## Abbreviations

BWH: Brigham and women’s hospital
C-CASA: Columbia classification algorithm of suicide assessment
cTAKES: clinical Text Analysis and Knowledge Extraction System
CUI: Concept unique identifier
DRG: Diagnosis-related group
EMRs: Electronic medical records
ICD: International classification of diseases
LMR: Longitudinal medical record
MedLEE: Medical language extraction and encoding system
MGH: Massachusetts general hospital
NLP: Natural language processing
PPV: Positive predictive values
RPDR: Research patient data registry
SNOMED-CT: Systemized nomenclature of medicine-clinical terms
UMLS: Unified medical language system
