# Supplementary material for: Screening pregnant women for suicidal behavior in electronic medical records: diagnostic codes vs. clinical notes processed by natural language processing
Source: BMC Med Inform Decis Mak. 2018 May 29;18:30. doi: 10.1186/s12911-018-0617-7 (PMC5975502; doi:10.1186/s12911-018-0617-7)
Supplement: Supplementary file 1 — Table S1. International Classification of Disease (ICD) codes and other diagnostic codes used to screen suicidal behavior. Table S2. Terms used to screen suicidal behavior in clinical notes. Table S3. Concept Unique Identifiers (CUIs) related to suicidal behavior. Table S4. Distributions of attributes of the Concept Unique Identifiers (CUIs) related to suicidal behavior among 1120 women. Table S5. International Classification of Disease (ICD) codes used to define psychiatric comorbidities. Table S6. Error analysis of false positive results from cTAKES to screen for suicidal behavior. (DOCX 36 kb) [file 12911_2018_617_MOESM1_ESM.docx]

**Table S1. International Classification of Disease (ICD) codes and other diagnostic codes used to screen suicidal behavior**

| **Type of codes** | **Codes** | **Strings** |
| --- | --- | --- |
| **Suicide (attempted)** | | |
| ICD-9 | E95* | Suicide and self-inflicted injury |
| ICD-10 | T14.91 | Attempted suicide NOS |
| ICD-10 | X71-X83 | Suicide (attempted)/Intentional self-harm |
| **Suicidal ideation** | | |
| ICD-9 | V62.84 |  |
| ICD-10 | R45.851 |  |
| **Suicide tendencies/suicide tendency - oncall** | | |
| LMR^1^ | LPA 407 |  |
| LMR^1^ | YMRA2 |  |
| **Poisoning by analgesics, antipyretics, and antirheumatics** | | |
| ICD-9 | 965 | Poisoning by analgesics, antipyretics, and antirheumatics |
| ICD-10 | T40.0X2 | Poisoning by opium, intentional self-harm |
| ICD-10 | T40.1X2 | Poisoning by heroin, intentional self-harm |
| ICD-10 | T40.3X2 | Poisoning by methadone, intentional self-harm |
| ICD-10 | T40.2X2 | Poisoning by other opioids, intentional self-harm |
| ICD-10 | T40.4X2 | Poisoning by other synthetic narcotics, intentional self-harm |
| ICD-10 | T40.6 02 | Poisoning by unspecified narcotics, intentional self-harm |
| ICD-10 | T40.692 | Poisoning by other narcotics, intentional self-harm |
| ICD-10: | T39.012 | Poisoning by aspirin, intentional self-harm |
| ICD-10 | T39.092 | Poisoning by salicylates, intentional self-harm |
| ICD-10 | T39.1X2 | Poisoning by 4-Aminophenol derivatives, intentional self-harm |
| ICD-10 | T39.2X2 | Poisoning by pyrazolone derivatives, intentional self-harm |
| ICD-10 | T39.312 | Poisoning by propionic acid derivatives, intentional self-harm |
| ICD-10 | T39.392A | Poisoning by other nonsteroidal anti-inflammatory drugs [NSAID], intentional self-harm, initial encounter |
| ICD-10 | T39.4X2 | Poisoning by antirheumatics, not elsewhere classified, intentional self-harm |
| ICD-10 | T39.8X2 | Poisoning by other nonopioid analgesics and antipyretics, not elsewhere classified, intentional self-harm |
| ICD-10 | T39.92 | Poisoning by unspecified nonopioid analgesic, antipyretic and antirheumatic, intentional self-harm |
| **Poisoning by psychotropic agents** | | |
| ICD-9 | 969 | Poisoning by psychotropic agents |
| ICD-10 | T43.202 | Poisoning by unspecified antidepressants, intentional self-harm |
| ICD-10 | T43.1X2 | Poisoning by monoamine-oxidase-inhibitor antidepressants, intentional self-harm |
| ICD-10 | T43.212 | Poisoning by selective serotonin and norepinephrine reuptake inhibitors, intentional self-harm, initial encounter |
| ICD-10 | T43.222 | Poisoning by selective serotonin reuptake inhibitors, intentional self-harm, initial encounter |
| ICD-10 | T43.022 | Poisoning by tetracyclic antidepressants, intentional self-harm, initial encounter |
| ICD-10 | T43.012 | Poisoning by tricyclic antidepressants, intentional self-harm, initial encounter |
| ICD-10 | T43.292 | Poisoning by other antidepressants, intentional self-harm, initial encounter |
| ICD-10 | T43.3X2 | Poisoning by phenothiazine antipsychotics and neuroleptics, intentional self-harm, initial encounter |
| ICD-10 | T43.4X2 | Poisoning by butyrophenone and thiothixene neuroleptics, intentional self-harm |
| ICD-10 | T43.502 | Poisoning by unspecified antipsychotics and neuroleptics, intentional self-harm |
| ICD-10 | T43.592 | Poisoning by other antipsychotics and neuroleptics, intentional self-harm |
| ICD-10 | T42.4X2 | Poisoning by benzodiazepines, intentional self-harm |
| ICD-10 | T40.7X2 | Poisoning by cannabis (derivatives), intentional self-harm |
| ICD-10 | T40.8X2 | Poisoning by lysergide [LSD], intentional self-harm |
| ICD-10 | T40.902 | Poisoning by unspecified psychodysleptics [hallucinogens], intentional self-harm |
| ICD-10 | T40.992 | Poisoning by other psychodysleptics [hallucinogens], intentional self-harm |
| ICD-10 | T43.602 | Poisoning by unspecified psychostimulants, intentional self-harm |
| ICD-10 | T43.612 | Poisoning by caffeine, intentional self-harm |
| ICD-10 | T43.622 | Poisoning by amphetamines, intentional self-harm |
| ICD-10 | T43.632 | Poisoning by methylphenidate, intentional self-harm |
| ICD-10 | T43.692 | Poisoning by other psychostimulants, intentional self-harm |
| ICD-10 | T43.8X2 | Poisoning by other psychotropic drugs, intentional self-harm |
| ICD-10 | T43.92X | Poisoning by unspecified psychotropic drug, intentional self-harm |
| **Poisoning by sedatives and hypnotics** | | |
| ICD-9 | 967 | Poisoning by sedatives and hypnotics |
| ICD-10 | T42.3X2 | Poisoning by barbiturates, intentional self-harm |
| ICD-10 | T42.6X2 | Poisoning by other antiepileptic and sedative-hypnotic drugs, intentional self-harm |
| ICD-10 | T42.72 | Poisoning by unspecified antiepileptic and sedative-hypnotic drugs, intentional self-harm |

^1^Longitudinal Medical Record (LMR), the ambulatory electronic medical record system used across Partners Healthcare System

**Table S2. Terms used to screen suicidal behavior in clinical notes**

- suicide, suicidal, suicidality,
- self harm, self-harm, harm to self, intentional self-harm, deliberate self-harm
- self poison, self-poison,
- passive SI
- kill herself, kill myself, killing herself, killing myself,
- self-injurious, self injurious, self injury, self-inflicted, self inflicted,
- self mutilation
- self cutting, deliberate self-cutting

**Table S3. Concept Unique Identifiers (CUIs) related to suicidal behavior**

- -C0038663    Suicide attempt Negated
- -C0038661    Suicide Negated
- C0438696     Suicidal
- C0563664     At risk for suicide
- C0038661     Suicide
- -C0424000    Feeling suicidal (finding) Negated
- C0038663     Suicide attempt
- -C0424366    Self-harm Negated
- C0424000     Feeling suicidal (finding)
- -C0085271    Self-Injurious Behavior Negated
- C0085271     Self-Injurious Behavior
- -C0438696    Suicidal Negated
- C0424366     Self-harm
- -C0522178    Thoughts of self harm Negated
- -C0563664    At risk for suicide Negated
- C1760428     Suicidal behavior
- -C1760428    Suicidal behavior Negated
- C0036601     Self Mutilation
- C1320313     Low risk of harm to self
- -C0455386    FH: Suicide Negated
- -C0582496    Suicidal intent Negated
- C0595861     Parasuicide
- -C0036601    Self Mutilation Negated
- C0522178     Thoughts of self harm
- C1276357     No suicidal thoughts
- C0204732     Suicide prevention
- C0582496     Suicidal intent
- -C0204732    Suicide prevention Negated
- -C1276357    No suicidal thoughts Negated
- C0455386     FH: Suicide

**Table S4. Distributions of attributes of the Concept Unique Identifiers (CUIs) related to suicidal behavior among 1,120 women**

| **Attributes of CUIs** | **Number of CUIs (N= 23,262)** | |
| --- | --- | --- |
|  | **n** | **%** |
| NegEx |  |  |
| Affirmed | 16389 | 70.45 |
| Negated | 6873 | 29.55 |
| *DocTimeRel* |  |  |
| Before | 2696 | 11.59 |
| After | 78 | 0.34 |
| Overlap | 20244 | 87.03 |
| Before/overlap | 243 | 1.04 |
| *Subject* |  |  |
| Patient | 22969 | 98.74 |
| Family member | 291 | 1.25 |
| Other | 2 | 0.01 |
| Null | 0 | 0.00 |

**Table S5. International Classification of Disease (ICD) codes used to define psychiatric comorbidities**

| **Psychiatric comorbidities** | **Type of codes** | **Codes** |
| --- | --- | --- |
| Depression | ICD-9 | 296.2, 296.3, 296.5, 296.8, 300.4, 309.0, 309.1, 311 |
|  | ICD-10 | F32.*, F33.*, F43.2 |
| Schizophrenia | ICD-9 | 295.* |
|  | ICD-10 | F20.* |
| Bipolar | ICD-9 | 296.0, 296.1, 296.4, 296.5, 296.6, 296.7, 296.8 |
|  | ICD-10 | F31.* |
| PTSD | ICD-9 | 309.81 |
|  | ICD-10 | F43.1 |
| Substance abuse | ICD-9 | 291.*, 292.*, 303.*, 304.*, 305.*, 648.3*, 655.5*, 965.0*, V65.42 |
|  | ICD-10 | F10.*, F11.*, F12.*, F14.1, F14.2, |
| Anxiety | ICD-9 | 293.84, 300.0, 300.2, 300.3, 300.7, 300.81, 308.2, 308.3, 309.2, 313.0 |
|  | ICD-10 | F40.*, F41.*, F42.*, F43.22 |

**Table S6. Error analysis of false positive results from cTAKES to screen for suicidal behavior**

| **cTAKES modules** | **Explanations** | **Examples** |
| --- | --- | --- |
| NegEx | Incorrectly identified negated terms as “affirmed | suicidal behavior: none  suicidal behavior: none reported  suicidal behavior: denied |
| *DocTimeRel* | Incorrectly treated history of suicidal behavior as current suicidal behavior | Suicide attempt/gesture: history of, hospitalized inpatient psych unit for suicide attempt in 1996 |
| *DocTimeRel* | Failure to handle hypothetical conditions that temporally are neither recent nor historical | If she has significant side effects from it such as lethargy/depression/irritability/suicidal thought, we will change it to LTG. |
| *Subject* | Incorrectly treated the suicidal behavior of patient’s father as patient’s | Pt also identifies strongly with father, who was often aggressive toward others and threatened suicide |
| Other | Failure to identify section titles that do not describe the behavior of patients | SUICIDAL BEHAVIOR HX OF SUICIDAL BEHAVIOR: |
